# Effect of glucocorticoid receptor gene polymorphisms on asthma phenotypes

**DOI:** 10.3892/etm.2012.809

**Published:** 2012-11-13

**Authors:** MICHAŁ PANEK, TADEUSZ PIETRAS, ARTUR FABIJAN, MACIEJ MIŁANOWSKI, ŁUKASZ WIETESKA, PAWEŁ GÓRSKI, PIOTR KUNA, JANUSZ SZEMRAJ

**Affiliations:** 1 Departments of Internal Medicine, Asthma and Allergy, Medical University of Lodz, Lodz 90-153; 2 Pneumology and Allergology, Medical University of Lodz, Lodz 90-153; 3 Student Research Group at the Department of Pneumology and Allergology, Medical University of Lodz, Lodz 90-153;; 4 Department of Medical Biochemistry, Medical University of Lodz, Lodz 92-215, Poland

**Keywords:** NR3C1 gene, glucocorticoid receptor, glucocorticoid receptor gene polymorphisms, single nucleotide polymorphism, restriction fragment length polymorphism, inflammation, asthma

## Abstract

The clinical presentation of asthma results from complex gene-gene and gene-environment interactions. The natural variability of the DNA sequence within the *NR3C1* gene affects the activity of glucocorticoid receptors (GCRs). The *NR3C1* gene is localized on chromosome 5q31–q32. The gene coding for the GCR comprises nine exons. The structural domains of the GCR determine the biological functions of the functional domains. The observed resistance to glucocorticosteroids and the normal metabolic profile of *Tth111*I single nucleotide polymorphism (SNP) carriers is due to the ER22/23EK polymorphism that is present in them. *Bcl*I polymorphism significantly affects the process of alternative *NR3C1* gene splicing and within that mechanism increases the sensitivity to glucocorticoids (GCs). A total of 451 subjects were enrolled in the present study, including 235 qualified to the group of bronchial asthma patients. A group of 216 healthy participants with no history of asthma or atopic conditions was qualified for the study. Genotyping was accomplished using the polymerase chain reaction-restriction fragment length polymorphism (PCR-RFLP) and PCR-high resolution melting (HRM) methods. No statistically significant differences were observed in the frequency of *Tth111*I, *Bcl*I and ER22/23EK polymorphisms of the *NR3C1* gene when comparing mild, moderate and severe asthma vs. the control group. Investigative analyses demonstrated statistically significant correlations for alleles and genotypes of *Tth111*I polymorphism of the *NR3C1* gene between healthy subjects and patients with severe asthma characterized by a control profile corresponding to an Asthma Control Test (ACT)™ score ≥20. It was established that only the *Tth111*I polymorphism of the *NR3C1* gene plays an important role in the pathogenesis of chronic bronchitis leading to the development of asthma with both allergic and non-allergic etiology.

## Introduction

Bronchial asthma is a chronic disease determined by multiple factors, with pathogenesis involving many cell types and substances secreted by them. The clinical presentation results from complex gene-gene and gene-environment interactions (1–4). One of the genes involved in the pathogenesis and clinical presentation of asthma is the *NR3C1* gene [(official symbol: *NR3C1* provided by the HUGO Gene Nomenclature Committee (HGNC); official full name: nuclear receptor subfamily 3, group C, member 1 (glucocorticoid receptor) provided by HGNC)], which encodes the glucocorticoid (GC) receptor (GCR) (5).

The *NR3C1* gene is localized on chromosome 5q31–q32 and consists of nine exons [National Center for Biotechnology Information (NCBI) Reference Sequence: NM_000176] (5,6). Exon 1 has seven basic transcriptional variants. Exons from 2 to 8 function as the core sequences of the *NR3C1* gene (7). Exon 9 determines two alternative splicing variants. The product of *NR3C1* gene expression is mRNA, which constitutes the basis for GCR isoforms formed as a result of alternative splicing (GCRα, GCRβ, GCRδ, GCRγ and GCR-P), among which only the α isoform is active (4,8,9). GCRβ is able to inhibit the signal pathway of isoform α and lead to impaired sensitivity to glucocorticosteroids.

**Figure 1.**
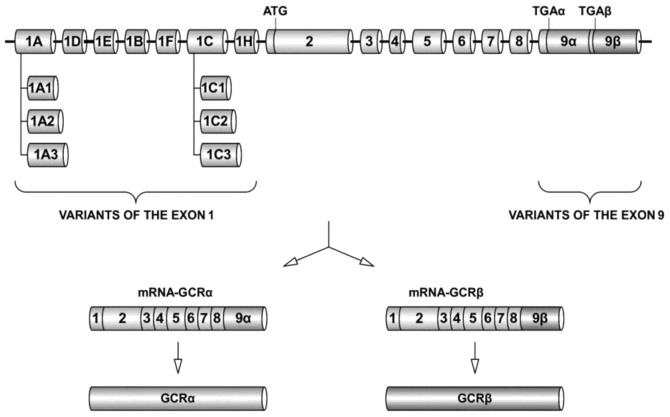
Organization and expression of the *NR3C1* gene. The gene coding for the glucocorticoid receptor (GCR) is made up of nine exons. Exon 1 has seven basic transcriptional variants (Ex1A, Ex1D, Ex1E, Ex1B, Ex1F, Ex1C and Ex1H). The core exons of the gene are those from Ex2 to Ex8. Exon 9 determines two alternative splicing variants: isoform α and isoform β of the GCR. Detailed description and relevant literature in the text. Authors’ own elaboration based on (8,15,16).

**Figure 2.**
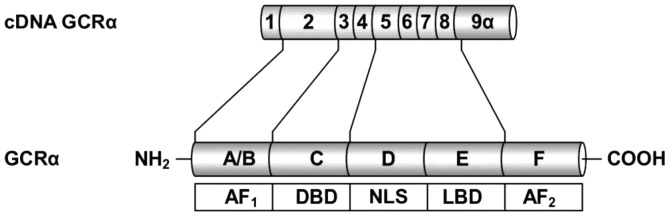
Structural and functional domains of the glucocorticoid receptor (GCR). The structural domains of the GCR: A/B, C, D, E and F determine the biological functions of the functional domains: activation function 1 (AF1), DNA-binding domain (DBD), nuclear localization sequence (NLS), ligand-binding domain (LBD) and AF2. Detailed description and relevant literature in the text. Authors’ own elaboration based on (4,8,9,15).

The GCR comprises 777 amino acids and five functional domains: activation function 1 (AF1), structural domain A/B; DNA-binding domain (DBD), structural domain C; nuclear localization sequence (NLS), structural domain D; ligand-binding domain (LBD), structural domain E; and activation function 2 (AF2), structural domain F (4,10–16). Figs. 1 and 2 show the organization of the *NR3C1* gene structure and relations between the structural and functional domains of the GCR.

The structure and biological activity of GCR domains is determined by the structure and nucleotide composition, among others, of the appropriate/corresponding exons of the *NR3C1* gene. The interactions of the GC/GCR complex and GC response element (GRE) may lead to selective activation or suppression of the target genes. The effect of GCs on the synthesis of proteins is determined by interactions with transcriptional enhancement sequences (GRE-positive) or transcriptional silencing sequences (GRE-negative) (4,9,10,14,17,18). The GCR interacts directly with expression coactivators and corepressors for numerous genes. Therefore, the GCR is a particularly important element determining appropriate patient responses to exogenous GCs, which are the most important anti-inflammatory agents for controlling the course of bronchial asthma (4,13,14,19–21).

**Figure 3.**
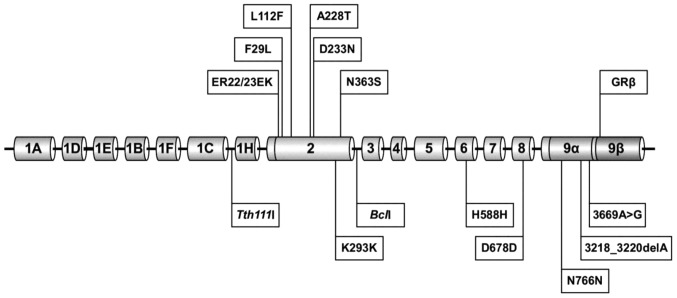
*NR3C1* gene polymorphisms. The upper part of the diagram presents single nucleotide polymorphisms (SNPs) causing amino acid variability. The lower part contains polymorphisms causing no changes in the glucocorticoid receptor structure. SNP positioning in the *NR3C1* according to the National Center for Biotechnology Information (NCBI) reference sequence: NM_000176. Detailed description and relevant literature in the text. Authors’ own elaboration based on (25,26).

Nucleotide changes in the studied gene DNA (single nucleotide polymorphisms; SNPs) may have a regulatory effect on expression and lead to changes in the RNA splicing process. Polymorphisms are responsible for modifications of the secondary and tertiary domain structures in the GCR, as well as for disturbances of transcription initiation and stability of the mRNA for the GCR (4,14,22–24). The locations of *NR3C1* gene polymorphisms are shown in Fig. 3.

Polymorphism *Tth111*I (rs10052957) is located in the area of the *NR3C1* gene promoter and localized in its transcript at position -13-6284. The SNP is situated in a large 27-kb intron just above the site of transcription initiation (between exons Ex1C and Ex1H) and 3807 bp above the first site where transcription in exon 2 begins. It causes a C>T substitution in the promoter region (25). It is considered that the observed resistance to glucocorticosteroids and the normal metabolic profile of *Tth111*I SNP carriers is due to ER22/23EK polymorphism that is present in them (24–27).

Polymorphism *Bcl*I (rs41423247) is located in intron 2 of the *NR3C1* gene and localized in its transcript at position 1184+646. Intron 2 (B) is situated between exons 2 and 3 of the *NR3C1* gene. It causes a C>G substitution in the promoter region. *Bcl*I SNP is localized 646 nucleotides above exon 2 of the *NR3C1* gene (6,28–30). *Bcl*I polymorphism significantly affects the process of alternative *NR3C1* gene splicing and within that mechanism increases the sensitivity to GCs. The above concerns both *Bcl*I CG and *Bcl*I GG (24,25,31).

Polymorphism ER22/23EK is located in exon 2 of the *NR3C1* and comprises two nucleotide transitions coupled with each other in codons 22 and 23 (24). A latent transition in codon 22 causes no amino acid substitution (G>A; position 198; codon GAG and GAA code for glutamic acid), whereas the transition in codon 23 causes substitution of arginine with lysine (G>A; AGG>AAG; position 200), which may consequently lead to a change in the tertiary structure of the GCR domain responsible for activation of transcription. Both polymorphisms are completely coupled with each other (9,24,32). The mechanism explaining GC resistance is the effect of ER22/23EK on the GCRα-A/GCRα-B ratio (24,27,16). The SNP correlates with increased production of the GCRα-B form, which results in decreased receptor capability of target gene transactivation (24,26,27).

The aim of the present study was to assess the effect of the selected *NR3C1* gene polymorphisms on the level of asthma control, as well as to identify in additional investigative studies their role in the determination of disease presentation phenotypes in particular patient groups.

## Materials and methods

### Patients and control subjects

Bronchial asthma patients were recruited from among those treated in the Department of Internal Medicine, Asthma and Allergy of N. Barlicki Memorial University Teaching Hospital No. 1, Lodz, Poland, Department of Pneumology and Allergology of N. Barlicki Memorial University Teaching Hospital and Specialist Outpatient Department of Pulmonary Diseases and Allergology at N. Barlicki Memorial University Teaching Hospital No. 1 of the Medical University of Lodz, Poland. Healthy volunteers from the general population were included in the study. They were selected on a random basis.

In total, 451 subjects were enrolled, including 235 qualified to the group of bronchial asthma patients. The inclusion criteria were: i) the patient’s informed consent to participate in the study was given and ii) appropriate spirometry results were obtained enabling the correct interpretation and diagnosis of asthma according to a report from The Global Initiative For Asthma (GINA) (1,2). Patients were excluded from the study on the basis of the following criteria: treatment with rifampicin, phenobarbital, phenytoin and/or ephedrine, and exacerbation of the disease due to infections. The bronchial asthma group comprised 62.6% (147) females and 37.4% (88) males. The mean age was 48.8 years [standard deviation (SD)±16.0], with a range of 19–82 years. Detailed parameters of the bronchial asthma patients are shown in Table I.

The control group comprised 216 healthy subjects. The subjects qualified to the control group met all the following criteria: i) no data from subjective and objective examinations confirming the presence of bronchial asthma, other pulmonary diseases, allergy, atopy and hypersensitivity to non-steroidal anti-inflammatory drugs (NSAIDs); ii) no spirometry results confirming airway obstruction; iii) negative results of skin prick tests with common allergens; iv) no first-degree relatives with asthma, allergy or atopy; and v) no treatment with rifampicin, phenobarbital, phenytoin and ephedrine.

The control group comprised 65.7% (142) females and 34.3% (74) males. The mean age was 45.7 years. (SD ±16.3), with a range of 18–85 years.

### Spirometry

Functional tests of the respiratory system were carried out according to the standards of the European Respiratory Society (ERS) and American Thoracic Society (ATS) (33).

Detailed descriptive statistics for the patient group and the control arm, including spirometry parameters, are shown in Table II.

### Asthma control test

The level of asthma control was assessed using the Asthma Control Test (ACT™), developed by Nathan *et al* and recommended by the GINA report. The bronchial asthma control level was calculated on the basis of the following patient results obtained by ACT: 0–19 points, no asthma control; 20–24 points, partially controlled asthma; and 25 points, well-controlled asthma (1,2,4,34).

### Skin-prick tests

Skin-prick tests were performed according to the guidelines of European Academy of Allergy and Clinical Immunology (EAACI) (35).

### Isolation of DNA

DNA was isolated from whole blood sampled into ethylenediaminetetraacetate (EDTA) by a spin column method using a QIAamp DNA Blood Mini kit (Qiagen, Hilden, Germany) according to the guidelines provided by the manufacturer.

### NR3C1 gene Tth111*I* polymorphism genotyping by the PCR-HRM method

The *Tth111*I polymorphism of the *NR3C1* gene was genotyped using the PCR-HRM method with application of the LightScanner^®^ 32 system (Idaho Technology, Inc., Salt Lake City, UT, USA). Exponential amplification of DNA segments for *Tth111*I polymorphism was carried out using forward (5′-GGA TGA ATC CCT ATC TGA GTG-3′) and reverse (5′-GGC CAC AAC AAT AAC CCA GTA-3′) primers according to standard PCR protocols. Primer binding to complementary DNA matrix sites was conducted at 58°C.

**Figure 4.**
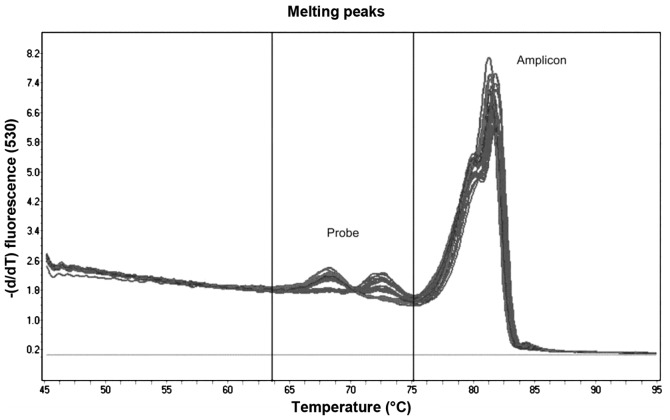
Melting curves obtained for the *Tth111*I SNP over the entire temperature range. Probe-target melting was observed between 64–75°C, while the amplicon melting occurred between 76–86°C.

The first stage of HRM analysis involved amplification of the investigated DNA fragment containing the analyzed *Tth111*I SNP on a 1:50 matrix using forward (5′-GCA GAG GTG GAA ATG AAG GTG-3′) and reverse (5′-GGA GTG GGA CAT AAA GCT ATG ACA-3′) primers, followed by denaturation and slow renaturation to form a heteroduplex. At the last stage, the mixture was subjected to precise denaturation in the presence of intercalating stain, and the identification of DNA fragments (*Tth111*I SNP) was based on the analysis of melting curves. The LightScanner^®^ High Sensitivity Master Mix (Idaho Technology, Inc.) was used for the reaction. This is a specialized master mix containing LCGreen Plus^®^ dye and internal temperature calibrators. The obtained product was subjected to internal control using a molecular probe with a C3-labeled carbon at the 3′-terminal portion [labeled, 3′-blocked oligonucleotide; 5′-ATG TAT TCA GAC TCA GTC AAG GCA AGG ACC T(SpcC3)-3′] (36–40). The selected SNP samples were verified by sequencing. Fig. 4 presents the melting curves for the probe and amplicon segment DNA fragments of the *Tth111*I SNP.

**Figure 5.**
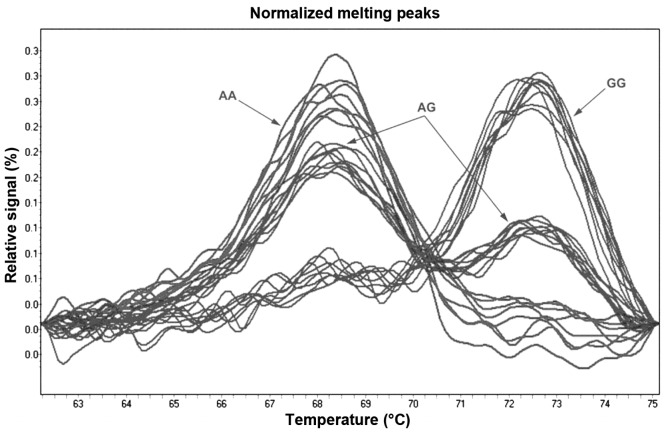
Normalized melting peaks for *Tth111*I polymorphism shown using unlabelled Luna Probes. The melting curves highlight homozygous mutation, heterozygous and wild-type.

Fig. 5 shows the probe normalization process with comparison of the denaturation curves and automatic identification of genotypes on the basis of differences in the melting temperature (GG and AA homozygotes) and denaturation curve shapes (AG heterozygotes) for *Tth111*I polymorphism.

**Figure 6.**
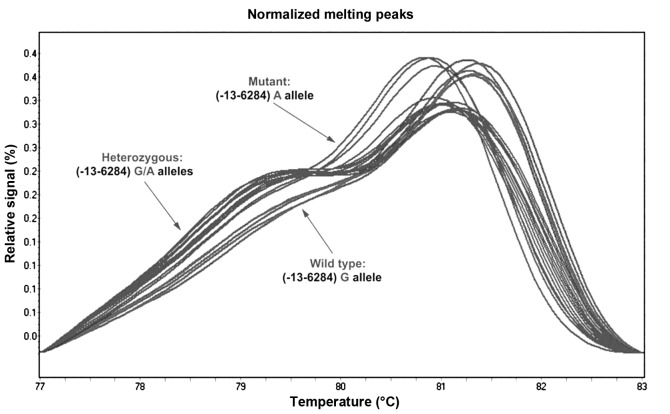
Normalized and temperature-shifted melting curves for *Tth111*I polymorphism. The melting curves depict homozygous mutation (AA), heterozygous (GA) and wild type (GG).

Fig. 6 shows the amplicon normalization process with comparison of the denaturation curves and automatic identification of genotypes on the basis of differences in the melting temperature (GG and AA homozygotes) and denaturation curve shapes (AG heterozygotes) for *Tth111*I polymorphism.

### Genotyping of BclI and ER22/23EK polymorphisms of the NR3C1 gene using the PCR-RFLP method

**Figure 7.**
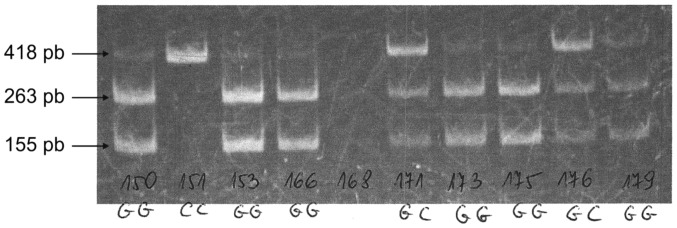
Detection of *Bcl*I polymorphism of the *NR3C1* gene using polymerase chain reaction-restriction fragment length polymorphism (PCR-RFLP) analyses.

Amplification of the DNA fragment containing *Bcl*I polymorphism of the *NR3C1* gene was conducted using primers with the following sequences: forward (5′-GAG AAA TTC ACC CCT ACC AAC-3′) and reverse (5′-AGA GCC CTA TTC TTC AAA CTG-3′), according to standard PCR protocols (41). Primer binding to complementary DNA matrix sites was conducted at 56°C. *Bcl*I restriction enzyme (Fermentas International Inc., Burlington, ON, Canada) was used for digestion of the amplification product containing the *Bcl*I polymorphism (41). Hydrolysis of the PCR product with the restriction enzyme was conducted for 24 h at 55°C. DNA fragments containing 263 and 151 bp identified as a set of representative, typical (wild type) alleles were obtained, as well as segments with 418, 263 and 155 bp. The RFLP product of 418-bp length was identified as a set of polymorphic alleles (41). RFLP products were separated by electrophoresis on 8% polyacrylamide gel, stained with ethidine bromide and observed in UV light (Image Master, Pharmacia Biotech, Uppsala, Sweden). Fig. 7 presents genotyping of the *Bcl*I polymorphism of the *NR3C1* gene using the RFLP method with *Bcl*I restrictase.

**Figure 8.**
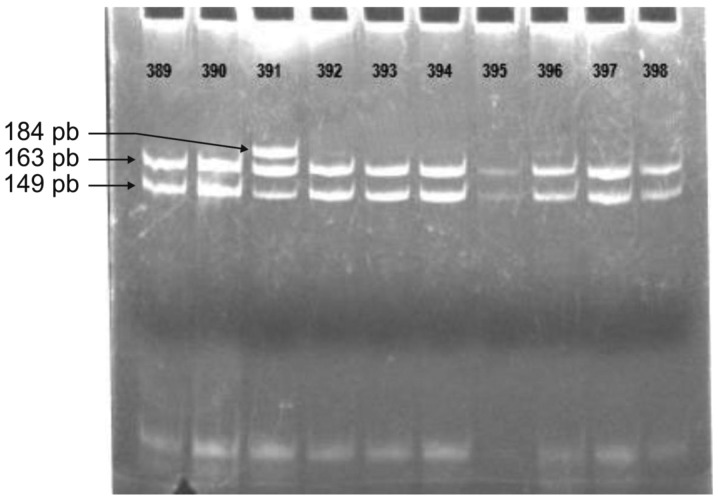
Detection of ER22/23EK polymorphism using polymerase chain reaction-restriction fragment length polymorphism (PCR-RFLP) analyses of the *NR3C1* gene.

Amplification of the DNA fragment containing ER22/23EK polymorphism of the *NR3C1* gene was conducted using primers with the following sequences: forward (5′-TGC ATT CGG AGT TAA CTA AAA-3′) and reverse (5′-ATC CCA GGT CAT TTC CCA TCA-3′) (41). Primer binding to complementary DNA matrix sites was conducted at 56°C. *Mnl*I restriction enzyme (Fermentas International) was used for digestion of the amplification product containing the ER22/23EK polymorphism (41). Hydrolysis of the PCR product with the restriction enzyme was conducted for 24 h at 37°C. DNA fragments with 149 and 163 bp (and shorter fragments containing 50, 49 and 35 bp) were obtained as a set of representative, typical (wild-type) alleles, whereas segments of 163 and 184 bp (and shorter fragments containing 50 and 49 bp) comprised a set of polymorphic alleles (41). RFLP products were separated by electrophoresis on 8% polyacrylamide gel, stained with ethidine bromide and observed in UV light (Image Master; Pharmacia Biotech). Fig. 8 presents the genotyping of the ER22/23EK polymorphism of the *NR3C1* gene using the RFLP method with *Mnl*I restrictase.

### Statistical analysis

Statistical calculations were carried out with a significance level of 0.05 using two-tailed tests. Correlations between *NR3C1* gene polymorphisms and asthma control levels were analyzed using the logistic regression model. The differences among the three genotypes were tested and the test for a linear trend was performed. The applied tests were based on the likelihood ratio. Statistical conclusions were based on tests for the linear trend due to their higher power. Bonferroni correction was also applied to the three polymorphisms tested. The odds ratios (ORs) as a measure of relative risk, and their 95% confidence intervals (CIs) were also calculated with a logistic regression model. The remaining correlations were tested using the Chi^2^-Pearson test. Furthermore, Hardy-Weinberg hypothesis concerning the equilibrium for allele distribution with respect to *Tth111*I, *Bcl*I and ER22/23EK polymorphisms of the *NR3C1* gene was tested using the tests available on-line at the website of the Institut für Humangenetik Technisches Universität München, Helmholtz Zentrum München, Deutsches Forschungszentrum für Gesundheit und Umwelt (42–46). Statistical analyses were performed using the R statistical software package (45).

### Approval of research review board

The study was approved by the local ethics committee (Consent of Research Review Board at the Medical University of Lodz, Poland, No RNN/133/09/KE). At the commencement of the study, the participants were invited to attend voluntarily. Prior to enrollment, written informed consent was obtained from all patients.

## Results

The frequencies of the genotypes of the *NR3C1* gene *Tth111*I polymorphism in the control group were estimated: the frequencies of GG, AG and AA were estimated as 0.297, 0.632 and 0.071, respectively. In the asthma population, the respective frequencies were 0.349, 0.534 and 0.117, and a higher frequency of the *Tth111*I polymorphism AA homozygote and lower frequency of the AG heterozygote in comparison with the control group were observed.

The estimated genotype frequencies of the *NR3C1* gene *Bcl*I polymorphism in the control group were determined; the frequencies of GG, GC and CC were 0.292, 0.592 and 0.116, respectively. In the asthma population, the respective frequencies were 0.282, 0.577 and 0.141, and a lower frequency of the *Bcl*I polymorphism GG homozygote and a higher frequency of the CC homozygote were observed in comparison with the control group.

The genotype frequencies of the *NR3C1* gene ER22/23EK polymorphism were estimated in the control group; the frequencies of GG, GA and AA were estimated as 0.935, 0.065 and 0.000, respectively. In asthma patients, the respective frequencies were observed to be 0.944, 0.056 and 0.000, and a higher frequency of the ER22/23EK polymorphism GG homozygote and a lower frequency of the GA heterozygote were observed in comparison with the control group. No AA homozygotes of the studied SNP were observed.

The consistency of genotype distribution in the control and asthma groups with Hardy-Weinberg equilibrium was demonstrated only for the ER22/23EK SNP of the *NR3C1* gene.

No statistically significant differences were observed in the frequencies of the *Tth111*I, *Bcl*I and ER22/23EK polymorphisms of the *NR3C1* gene when mild, moderate, and severe asthma were compared against the control group (p>0.05).

Statistically significant differences were identified in the distribution of *Tth111*I polymorphism of the *NR3C1* gene between asthma patients stratified according to asthma control levels based on ACT™ scores (<20 vs. ≥20; p<0.05). Table III shows the distribution of genotype frequencies for *Tth111*I polymorphism among asthma patients taking into consideration the disease control level based on ACT™ scores (<20 vs. ≥20).

No statistically significant differences were noted in the distribution of genotype frequencies for *Bcl*I and ER22/EK23 polymorphisms of the *NR3C1* gene between the populations of asthma patients taking into consideration the disease control level based on ACT™ score (<20 vs. ≥20; p>0.05). The results in this study did not confirm previous observations on the role of *Bcl*I polymorphism of *NR3C1* gene in the pathogenesis of asthma. A *Bcl*I SNP pilot study was carried out on a small number of patients and controls. This confirms the need for genetic testing in a larger number of cases (47). It is worth noting that the current study highlights the lack of association between polymorphic forms ER22/23EK of *NR3C1* gene in the pathogenesis of asthma (48).

In view of the results obtained for *Tth111*I polymorphism of the *NR3C1* gene in asthma patients, additional exploration analyses were carried out within that group. The trends for potential correlations within the patient population were identified by considering the disease control level according to ACT™ score and the disease severity according to GINA criteria. The correlations between the frequencies of alleles and genotypes in the patient population and in the controls were evaluated.

Additional analyses addressing the frequencies of *NR3C1* gene *Tth111*I polymorphism alleles and genotypes demonstrated statistically significant correlations between healthy controls and patients with asthma characterized by a control profile corresponding to an ACT™ score ≥20, as shown in Table IV.

Exploration analyses demonstrated statistically significant correlations for alleles and genotypes of the *Tth111*I polymorphism of the *NR3C1* gene between healthy subjects and patients with severe asthma characterized by a control profile corresponding to an ACT™ score ≥20, between healthy subjects and patients with non-allergic severe asthma characterized by a control profile corresponding to an ACT™ score ≥20, and between healthy subjects and patients with allergic severe asthma characterized by a control profile corresponding to an ACT™ score ≥20, as shown in Tables V, VI and VII, respectively.

## Discussion

The present study concerns a new aspect of the role of genetic factors in the complex etiopathogenesis of chronic bronchitis and bronchial asthma, which approaches asthma in a multidimensional manner. The study proposes a novel strategic approach for assessing the effect of hereditary variables in the disease. The first dimension comprises genetic factors (gene polymorphisms); the second, the severity of the disease (assessed on the basis of the GINA report criteria: mild, moderate or severe); and the third, asthma symptom control level (assessed individually by the patients on the basis of ACT™ score).

It should be emphasized that the concept of a multidimensional approach to disease was first introduced when the system of classification of mental and behavioral disturbances, DSM IV, was being developed. At that time, the multidimensional approach was applied to the categorization of schizophrenia symptoms during the development of the positive and negative syndrome scale (PANSS) (46,49). This approach is increasingly widely used in the classification of somatic disorders, as exemplified by the updated version of the GOLD 2011 report (49,50).

The studied populations were imbalanced with respect to *Bcl*I and *Tth111*I polymorphisms, which may be explained by the lethal character of changes in nucleotide variances of the analyzed *NR3C1* gene. Another potential reason for disequilibrium in genotype distribution may be non-random selection of sexual partners. As partners are selected on the basis of certain characteristics, the distribution of genotypes in the population deviates from equilibrium.

No differences in the frequencies of *Bcl*I and ER22/23EK polymorphisms of the *NR3C1* gene between asthma patients and healthy controls were observed. No correlation was found between the frequencies of *Bcl*I and ER22/23EK *NR3C1* gene polymorphisms and asthma control level in the patient group.

However, the study demonstrated a correlation between the presence of the *Tth111*I polymorphism of the *NR3C1* gene and a specific profile of asthma control according to ACT™. Additionally, correlations were demonstrated between the presence of the *Tth111*I *NR3C1* gene polymorphism in the patient group and the disease severity and higher asthma control level based on ACT™ scores. Correlations were noted between the frequency of *Tth111*I SNP alleles in the population of asthma patients with disease control profiles corresponding to an ACT™ score ≥20 and healthy controls.

Allele A of the *Tth111*I *NR3C1* gene polymorphism was demonstrated to be an important factor correlating with the risk of development for specific disease phenotypes (severe asthma with ACT™ score ≥20, severe non-allergic asthma with ACT™ score ≥20 and severe allergic asthma with ACT™ score ≥20). The expression ratio of two A alleles of the analyzed SNP was found to be the determinant most strongly correlated with higher control levels of severe asthma, both allergic and non-allergic. For the above analyses, high statistical significance levels were observed, which confirms the role of *Tth111*I *NR3C1* gene polymorphism in the pathogenesis of specific disease phenotypes.

Therefore, it appears that the correct approach to the analysis of the role of SNPs in the etiology of pulmonary diseases is a multifactorial interpretation of the function of *NR3C1* gene polymorphisms in the pathogenesis of obstructive syndromes. According to this approach to asthma, the significance of only one of the analyzed SNPs (*Tth111*I) has been demonstrated. The site of the *Tth111*I polymorphism is located in the intron close to the initiation site and 3807 pb above the first site where transcription starts in exon 2. It causes G>A substitution in the promoter region, and is also associated with ER22/23EK SNP localized in exon 2 of the *NR3C1* gene (25,28). It is noteworthy that a G-13-6284A type SNP in this *NR3C1* gene domain may affect, in combination with ER22/23EK, structural changes in the A/B region of the GCR and functional changes within the AF1 functional domain, which influences the GCR activity and enables interaction with numerous transcriptional factors (4,6,24,27,29).

The current study is an important part of a campaign aimed at the prevention of asthma (especially severe asthma) as well as early diagnosis, planning of therapy adjusted to the patient’s requirements, and improvement of care for patients with uncontrolled symptom profiles. Thus, there are important prerequisites to develop a research technique using *Tth111*I as a diagnostic marker of cases requiring individual glucocorticosteroid therapy. Evaluation of its applicability in this area requires further investigation.

In conclusion, the concept of asthma endotype separation as a new approach to classification of disease occurs more often in genetic screening according to data provided by the NCBI. Intermediate phenotypes of asthma are morphologically or functionally defined. This approach is based on the molecular mechanism or response to treatment.

It was established that the *Tth111*I polymorphism of the *NR3C1* gene plays an important role in the pathogenesis of chronic bronchitis leading to the development of asthma with both allergic and non-allergic etiology. This SNP correlates significantly with the severe asthma phenotype characterized by a control profile corresponding to an ACT™ test score ≥20.

Polymorphism *Tth111*I of the *NR3C1* gene differentiates asthma patients according to the symptom control level based on the ACT™ test score into two subpopulations (ACT™ score <20 vs. ACT™ score ≥20).

In conclusion, homozygote AA *Tth111*I correlates with a more moderate course of the disease and a higher control level of its symptoms.

## Figures and Tables

**Table I.** Characteristics of patients with asthma, including the degree of disease severity.

Classification of asthma according to severity	N	%
Asthma	235	100.00
Non-severe	151	64.26
Severe	84	35.74
Chronic mild	35	14.90
Chronic moderate	116	49.36
Chronic severe	84	35.74

**Table II.** Descriptive statistics of the analyzed parameters in control and case (general asthma population) groups in the present study.

Parameter	Group	
	Controls	Asthma patients
Number		
n	216	235
M/F (%)	74 (34.3)/142 (65.7)	88 (37.4)/147 (62.6)
Age (years)		
Mean	45.7	48.8
SD	16.3	16.0
Median	47.0	51.0
Mode	23.0	52.0
FEV_1_ (liters)		
Mean	3.0	2.2
SD	0.8	0.9
Median	2.9	2.2
Mode	2.7	2.4
FEV_1_ (%)		
Mean	96.1	72.7
SD	12.6	19.8
Median	96.0	74.0
Mode	94.5	90.0
FEV_1_/FVC (%)		
Mean	79.0	66.3
SD	6.1	12.0
Median	78.6	66.9
Mode	78.5	70.7
FVC (litres)		
Mean	3.8	3.3
SD	1.0	1.1
Median	3.6	3.2
Mode	3.5	2.3
FVC (%)		
Mean	102.7	91.4
SD	14.9	17.5
Median	101.0	93.0
Mode	101.0	93.0

M, male; F, female; SD, standard deviation; FEV_1_, forced expiratory volume in 1 sec; FVC, forced vital capacity.

**Table III.** Comparative analysis of *NR3C1* gene *Tth111*I polymorphism frequency among asthma patients taking into consideration the disease control level based on Asthma Control Test (ACT)™ scores (<20 vs. ≥20).

	[AA] vs. [AG]	[AA] vs. [GG]	[AA] vs. [AG+GG]
OR	0.275	0.344	0.301
CI	0.114–0.663	0.138–0.858	0.129–0.703
Chi^2^	8.91	5.46	8.36
P-value	0.008	0.057	0.011

OR, odds ratio; CI, confidence interval.

**Table IV.** Comparative analysis of *NR3C1* gene *Tth111*I polymorphism frequency in healthy controls vs. patients with asthma characterized by a control profile corresponding to an ACT™ score ≥20.

	Healthy controls n (expected)	Asthma ACT™ score ≥20 n (expected)	OR	CI	Chi^2^	P-value
GG	64 (81.04)	33 (31.84)				
AG	136 (101.92)	44 (46.32)				
AA	15 (32.04)	18 (16.84)				
[AG] vs. [GG]			0.270	0.125–0.579	12.25	0.00047
[AA] vs. [GG]			0.430	0.192–0.960	4.35	0.03699
[AA] vs. [AG + GG]			0.321	0.154–0.668	9.93	0.00163

The tests for association are adapted from Sasieni (42,43). OR, odds ratio; CI, confidence interval; ACT™, Asthma Control Test.

**Table V.** Comparative analysis of *NR3C1* gene *Tth111*I polymorphism frequency in healthy controls vs. patients with severe asthma characterized by a control profile corresponding to an ACT™ score ≥20.

	Healthy controls n (expected)	Severe asthma ACT™ score ≥20 n (expected)	OR	CI	Chi^2^	P-value
GG	64 (81.04)	8 (5.76)				
AG	136 (101.92)	6 (10.48)				
AA	15 (32.04)	7 (4.76)				
[AG] vs. [GG]			0.095	0.028–0.318	19.87	8.23e-06
[AA] vs. [GG]			0.268	0.084–0.854	5.39	0.02028
[AA] vs. [AG+GG]			0.150	0.053–0.428	15.72	0.00007

The tests for association are adapted from Sasieni (42,43). OR, odds ratio; CI, confidence interval; ACT™, Asthma Control Test.

**Table VI.** Comparative analysis of *NR3C1* gene *Tth111*I polymorphism frequency in healthy controls vs. patients with severe non-allergic asthma characterized by control profile corresponding to ACT™ score ≥20.

	Healthy controls n (expected)	Severe non-allergic asthma ACT™ score ≥20 n (expected)	OR	CI	Chi^2^	P-value
GG	64 (81.04)	4 (2.25)				
AG	136 (101.92)	1 (4.50)				
AA	15 (32.04)	4 (2.25)				
[AG] vs. [GG]			0.028	0.003–0.263	22.21	2.440e-06
[AA] vs. [AA + GG]			0.094	0.023–0.386	15.62	0.00008

The tests for association are adapted from Sasieni (42,43). OR, odds ratio; CI, confidence interval; ACT™, Asthma Control Test.

**Table VII.** Comparative analysis of *NR3C1* gene *Tth111*I polymorphism frequency in healthy controls vs. patients with severe allergic asthma characterized by a control profile corresponding to an ACT™ score ≥20.

	Healthy controls n (expected)	Severe allergic asthma ACT™ score ≥20 n (expected)	OR	CI	Chi^2^	P-value
GG	64 (81.04)	4 (3.52)				
AG	136 (101.92)	5 (5.96)				
AA	15 (32.04)	3 (2.52)				
[AG] vs. [GG]			0.184	0.040–0.847	5.75	0.01648
[AA] vs. [AG + GG]			0.225	0.055–0.920	5.06	0.02453

The tests for association are adapted from Sasieni (42,43). OR, odds ratio; CI, confidence interval; ACT™, Asthma Control Test.

## Abbreviations:

ACT™: Asthma Control Test
AF: activation function
ATS: American Thoracic Society
CI: confidence interval
DBD: DNA-binding domain
EAACI: European Academy of Allergy and Clinical Immunology
EDTA: ethylenediaminetetraacetic acid
ERS: European Respiratory Society
FEV_1_: forced expiratory volume in 1 sec
FVC: forced vital capacity
GCR: glucocorticoid receptor
GCs: glucocorticoids
GINA: Global Initiative For Asthma
GRE: glucocorticoid response element
HGNC: HUGO Gene Nomenclature Committee
HRM: high resolution melting
LBD: ligand-binding domain
mRNA: messenger RNA
NCBI: National Center for Biotechnology Information
NLS: nuclear localization sequence
*NR3C1*: nuclear receptor subfamily 3, group C, member 1 (glucocorticoid receptor)
NSAIDs: non-steroidal anti-inflammatory drugs
OR: odds ratio
SD: standard deviation
PCR: polymerase chain reaction
RFLP: restriction fragment length polymorphism
SNP: single nucleotide polymorphism.
